# Clinical scoring systems, molecular subtypes and baseline [^18^F]FDG PET/CT image analysis for prognosis of diffuse large B-cell lymphoma

**DOI:** 10.1186/s40644-024-00810-8

**Published:** 2024-12-18

**Authors:** Zhuxu Sun, Tianshuo Yang, Chongyang Ding, Yuye Shi, Luyi Cheng, Qingshen Jia, Weijing Tao

**Affiliations:** 1 Department of Nuclear Medicine, The Affiliated Huaian No.1 People’s Hospital of Nanjing Medical University, Huai’an, Jiangsu China; 2 Department of Nuclear Medicine, The First Affiliated Hospital of Nanjing Medical University, Nanjing, Jiangsu China; 3 Department of Hematology, The Affiliated Huaian No.1 People’s Hospital of Nanjing Medical University, Huai’an, Jiangsu China; 4 State Key Laboratory of Medicinal Chemical Biology, College of Pharmacy, Tianjin Central Hospital of Gynecology Obstetrics, Tianjin Key Laboratory of Human Development and Reproductive Regulation, Nankai University, Tianjin, China

**Keywords:** Diffuse large B-cell lymphoma, FDG PET/CT, Clinical scoring systems, Molecular subtypes, Prognosis

## Abstract

Diffuse large B-cell lymphoma (DLBCL) is a highly heterogeneous hematological malignancy resulting in a range of outcomes, and the early prediction of these outcomes has important implications for patient management. Clinical scoring systems provide the most commonly used prognostic evaluation criteria, and the value of genetic testing has also been confirmed by in-depth research on molecular typing. [^18^F]-fluorodeoxyglucose positron emission tomography / computed tomography ([^18^F]FDG PET/CT) is an invaluable tool for predicting DLBCL progression. Conventional baseline image-based parameters and machine learning models have been used in prognostic FDG PET/CT studies of DLBCL; however, numerous studies have shown that combinations of baseline clinical scoring systems, molecular subtypes, and parameters and models based on baseline FDG PET/CT image may provide better predictions of patient outcomes and aid clinical decision-making in patients with DLBCL.

## Background

Diffuse large B-cell lymphoma (DLBCL) is the most common non-Hodgkin lymphoma and accounts for 30% of all lymphomas. Although 60%−70% of patients with newly diagnosed DLBCL can be cured using the traditional standard therapy combining rituximab, cyclophosphamide, doxorubicin, vincristine, and prednisone (R-CHOP), up to 30%−40% of patients will have disease that is refractory to this treatment or will have a relapse after an initial response [1–6]. Patients with refractory disease have a poor prognosis after salvage chemotherapy, but their outcomes may be greatly improved following Pola-R-CHP, which was approved by Food and Drug Administration (FDA) in 2023, immunotherapy or targeted therapies [5–7]. It is therefore necessary to construct an accurate model for predicting patient outcomes to enable early risk stratification and optimal treatment decisions for patients with DLBCL [8].

Clinical predictive indicators have been widely used for assessing DLBCL prognosis. Additionally, baseline FDG PET/CT plays an increasingly important role in predicting DLBCL outcomes. Since 2014, the International Conference on Malignant Lymphoma imaging consensus guidelines has recognized the use of FDG PET/CT for evaluating glucose metabolism in lymphoma lesions [9–12]. Other FDG-PET-derived parameters, such as metabolic tumor volume (MTV), total lesion glycolysis (TLG), and maximal distance between two farthest lesions (Dmax) may also predict DLBCL outcomes [13].

Numerous studies have used novel indicators and risk factors to construct new models to predict DLBCL progression. This review focuses on the important clinical scoring systems, molecular subtypes, and FDG PET/CT parameters related to DLBCL prognosis, to guide the selection of treatment regimens after the prediction of DLBCL outcomes.

### Clinical scoring systems

The International Prognostic Index (IPI) clinical scoring system has been widely used for risk stratification and to select rational therapeutic strategies in patients with DLBCL since 1993 [14, 15]. IPI versions have been updated to stratify patient prognosis along with changes in DLBCL treatment methods, including the revised IPI (R-IPI), National Comprehensive Cancer Network IPI (NCCN-IPI), central nervous system IPI (CNS-IPI), and age-adjusted IPI (aa-IPI) (Table 1).

**Table 1:** Summary of the clinical scoring systems mentioned in this study

Clinical scoring systems	Release year	Factors	Score	Total score: risk group
IPI	1993	Age > 60 ys	1	0-1: Low 2: Low-intermediate 3: High-intermediate 4-5: High
		ECOG PS ≥ 2	1	
		LDH > normal	1	
		Extranodal involvement > 1	1	
		Ann Arbor stage III/IV	1	
aa-IPI	1993	Age ≤ 60 ys		0: Low 1: Low-intermediate 2: High-intermediate 3: High
		ECOG PS ≥ 2	1	
		LDH > normal	1	
		Ann Arbor stage III/IV	1	
R-IPI	2006	Age > 60 ys	1	0: Very good 1-2: Good 3-5: Poor
		ECOG PS ≥ 2	1	
		LDH > normal	1	
		Extranodal involvement > 1	1	
		Ann Arbor stage III/IV	1	
NCCN-IPI	2014	Age, ys		0-1: Low 2-3: Low-intermediate 4-5: High-intermediate 6-8: High
		> 40 to ≤ 60	1	
		> 60 to ≤ 75	2	
		> 75	3	
		LDH, normalized		
		> 1 to ≤ 3	1	
		> 3	2	
		Ann Arbor stage III-IV	1	
		Extranodal disease	1	
		Performance status ≥ 2	1	
CNS-IPI	2016	Kidney and/or adrenal glands involved	1	0-1: Low 2-3: intermediate 4-6: High
		Age > 60 ys	1	
		LDH > normal	1	
		ECOG PS > 1	1	
		Ann Arbor stage III/IV	1	
		Extranodal involvement > 1	1	
IBPS	2023	SII ≥ 1109.90	1	0-1: Low 2-5: High
		PNI ≥ 42.55	1	
		mGPS		
		C-reactive protein ≤ 10 mg/L	0	
		C-reactive protein > 10 mg/L		
		Albumin ≥ 35 g/L	1	
		Albumin < 35 g/L	2	

*IPI* International Prognostic Index, *ECOG* Eastern Cooperative Oncology Group, *aa-IPI* age-adjusted IPI, *R-IPI* revised IPI, *NCCN-IPI* National Comprehensive Cancer Network IPI, *CNS-IPI* central nervous system IPI, *IBPS* Inflammation-Based Prognosis Score, *SII* Systemic immune-inflammation index, *PNI* prognostic nutrition index, *mGPS* modified Glasgow prognostic score

### IPI and aa-IPI

The IPI stratifies DLBCL into four discrete risk categories (low, low-intermediate, high-intermediate and high) with five clinical characteristics: age, lactate dehydrogenase level, number of extra-nodal sites, Ann Arbor stage, and Eastern Cooperative Oncology Group (ECOG) performance status; however, its stratified prognostic ability has been greatly reduced by gradual changes in DLBCL treatment methods [15–17].

The aa-IPI was developed for patients aged ≤ 60 years, who have notably different outcomes from older patients, and thus this is the age limit for the most-intensive experimental treatments for non-Hodgkin lymphoma. The aa-IPI involves three adverse prognostic factors: disease stages III-IV, high lactate dehydrogenase level, and ECOG performance status ≥ 2 [18].

### R-IPI

Since the late 1990s, rituximab (R) added to CHOP for DLBCL has significantly improved survival among all risk groups; however, the IPI discrimination ability has declined, especially among higher-risk patients. The R-IPI was therefore developed to risk-stratify DLBCL patients treated with R-CHOP [15, 16, 19] into three risk groups: low [0], intermediate [1, 2], and high [3–5, 16]. Sehn et al. used the R-IPI to identify three distinct prognostic groups with very good (94%), good (79%), or poor (55%) overall survival (OS) (*P* < 0.001) [14]. More precise grouping can help doctors to balance efficacy against excessive toxicity.

### NCCN-IPI

Neither the IPI nor the R-IPI can identify risk groups with < 50% chance of survival (4-year OS: IPI 59%, R-IPI 55%) [14, 20]. Pooled data showed a 5-year OS of approximately 50% in the IPI high-risk group, and an enhanced NCCN-IPI was therefore constructed to identify the above subgroups [19]. Unlike the IPI system, the NCCN-IPI regarded bone marrow, CNS, liver/gastrointestinal tract, and lung lesions as risk factors [21]. The IPI, R-IPI, and NCCN-IPI gave 5-year OS estimates with accuracy rates of 54%-88%, 61%-93%, and 49%-92%, respectively. The NCCN-IPI may be the best-performing scoring system, with similar ability of the R-IPI for discerning subgroups with favorable long-term survival and better ability than the IPI for detecting a less-heterogeneous high-risk group [15, 20, 22].

### CNS-IPI

CNS infiltration occurs in 2%-10% of DLBCLs patients, and the CNS-IPI is developed for cases involving CNS relapse. The prognostic score includes IPI risk factors and involvement of the kidney and/or adrenal glands [23]. The CNS-IPI-predicted CNS relapse rates were 0.0%, 0.8%, and 13.8% for patients with low, intermediate, and high risk, respectively [24]. The CNS-IPI has thus been proposed as a prognostic tool to improve prospective validation and guide therapy [24, 25].

Compared with CNS-IPI alone, the combination model of CNS-IPI, such as the model based on high CNS-IPI score and ABC/unclassified cell of origin (COO) or based on CNS-IPI and the model incorporating images and clinical variables, could identified the high-risk population with a higher 2-year CNS-relapse probability (15.2% or 17.1% vs 8.9%) [26, 27].

### IBPS and R/R-IPI

Scoring systems other than IPI-based systems also exist. The Inflammation-Based Prognosis Score (IBPS) was constructed from the systemic immune inflammation index, prognostic nutrition index, and modified Glasgow prognostic score, and generated C-indices for OS in training and validation cohorts of 0.844 and 0.828, respectively [28]. The relapsed/refractory-IPI(R/R-IPI) was constructed for relapsed/refractory DLBCL patients, using only age and front-line time to progression, with good C-indices in discovery (0.67) and validation sets (0.64, 0.68). This study provided a robust method with readily available clinical details to identify patients that should be considered for immediate treatment with the complex and costly chimeric antigen receptor T-cell therapy [29, 30].

The addition of new clinical indicators has been proposed to improve the predictive ability of existing scoring systems, including low serum cholesterol, uric acid, and apolipoprotein A-I, absolute lymphocyte/monocyte ratio, red blood cell distribution width, platelet count, and beta-2 microglobulin level [31–33].

### Molecular subtypes

Although the IPI is easy to apply in clinical practice, it does not fully account for disease heterogeneity [8]. Gene expression profiling has identified DLBCL subgroups (activated B-cell-like [ABC], germinal-center B-cell-like [GCB], and unclassified) according to the cells of origin. ABC DLBCL is characterized by B-cell-receptor dependence, constitutive nuclear factor-κB activation, and interferon regulatory factor4 (IRF4) /MUM1 multiple myeloma oncogene1 (MUM1) expression, while GCB DLBCL is characterized by CD10 expression and BCL2 rearrangements [8, 34, 35]. Compared with GCB, ABC DLBCL has higher risk of relapse and inferior outcome following R-CHOP [35, 36].

The appropriate treatments based on the subtype classification can improve prognosis. Different subclassifications requiring fluorescence in situ hybridization testing to identify MYC, BCL2, and BCL6 rearrangements have been introduced to identify patients with increased risk profiles [37, 38]. MYC translocation is a strong adverse prognostic factor related to inferior OS and progression-free survival (PFS) [35]. Li et al. recommended bromodomain and extra-terminal protein family inhibitor therapy, either alone or in combination with other drugs, to improve the prognosis of patients with MYC expression [39].

Previous studies designated the 5%–15% of DLBCL cases with MYC, BLC2, and/or BCL6 translocations as DHL (MYC/BLC2, MYC/BCL6) and THL (MYC/BLC2/BCL6), respectively [40–43]. However, the 2022 World Health Organization (WHO) and International Consensus Classification (ICC) recommendations re-categorized MYC/BCL6 as “DLBCL, not otherwise specified” and MYC/BCL2 and MYC/BLC2/BCL6 as “DLBCL/high-grade B-cell lymphoma-MYC/BCL2” [34, 40, 41]. Based on these new classifications, a multicenter, retrospective study including 220 patients with DLBCL revealed that MYC/BCL6 patients had superior and longer OS than patients with MYC/BCL2-rearrangements and THL, and treatment intensification was associated with next treatment time and OS in patients with MYC/BCL2 and THL but no improvement in MYC/BCL6 patients [41]. More clinical trials are needed to confirm the optimal classification for DLBCL prognosis.

As mentioned above, molecular subclassification might predict clinical outcomes of current therapeutic strategies, with specific phenotypes enabling the development of precision therapies [35, 36]. A study of 412 patients with DLBCL identified 14 metabolism-associated genes characteristic of the immunosuppressive microenvironment and associated with prognosis. The resulting metabolism-associated prognosis risk model may facilitate personalized treatment strategies and provide the basis for further studies of metabolism-associated genes and the immune microenvironment [42].

R-CHOP therapy may be extended to include personalized treatment with agents targeting genes for DLBCL. A randomized phase II trial reported that R-CHOP-X including targeted Bruton’s tyrosine kinase inhibitors (ibrutinib), histone deacetylase inhibitors, demethylating agents (decitabine), and lenalidomide based on mutated MCD, BN2, EZB, TP53, and N1, resulted in significantly higher 2-year PFS and OS rates than R-CHOP [43]. Wang's et al. study demonstrated that high cyclin D2 (CCND2) expression in ABC DLBCL was an independent prognostic indicator of PFS, potentially promoting further research on CCND2 inhibition and R-CHOP combination therapy [44]. Acylglycerol kinase inhibitors represent another possible approach to enhance the efficacy of venetoclax (a highly selective BCL-2 inhibitor) [45], while other targeted agents include the anti-CD79b antibody–drug conjugate polatuzumab vedotin, and anti-CD19 chimeric antigen receptor T-cell products [37].

### FDG PET/CT parameters

FDG PET/CT is an essential screening tool for DLBCL because it can reflect differential glycolytic activity between lesions and healthy tissue [46, 47]. Baseline PET metrics have demonstrated prognostic value in DLBCL in many studies, including a phase III clinical trial of obinutuzumab plus CHOP chemotherapy (GOYA) [48].

**Fig. 1:**
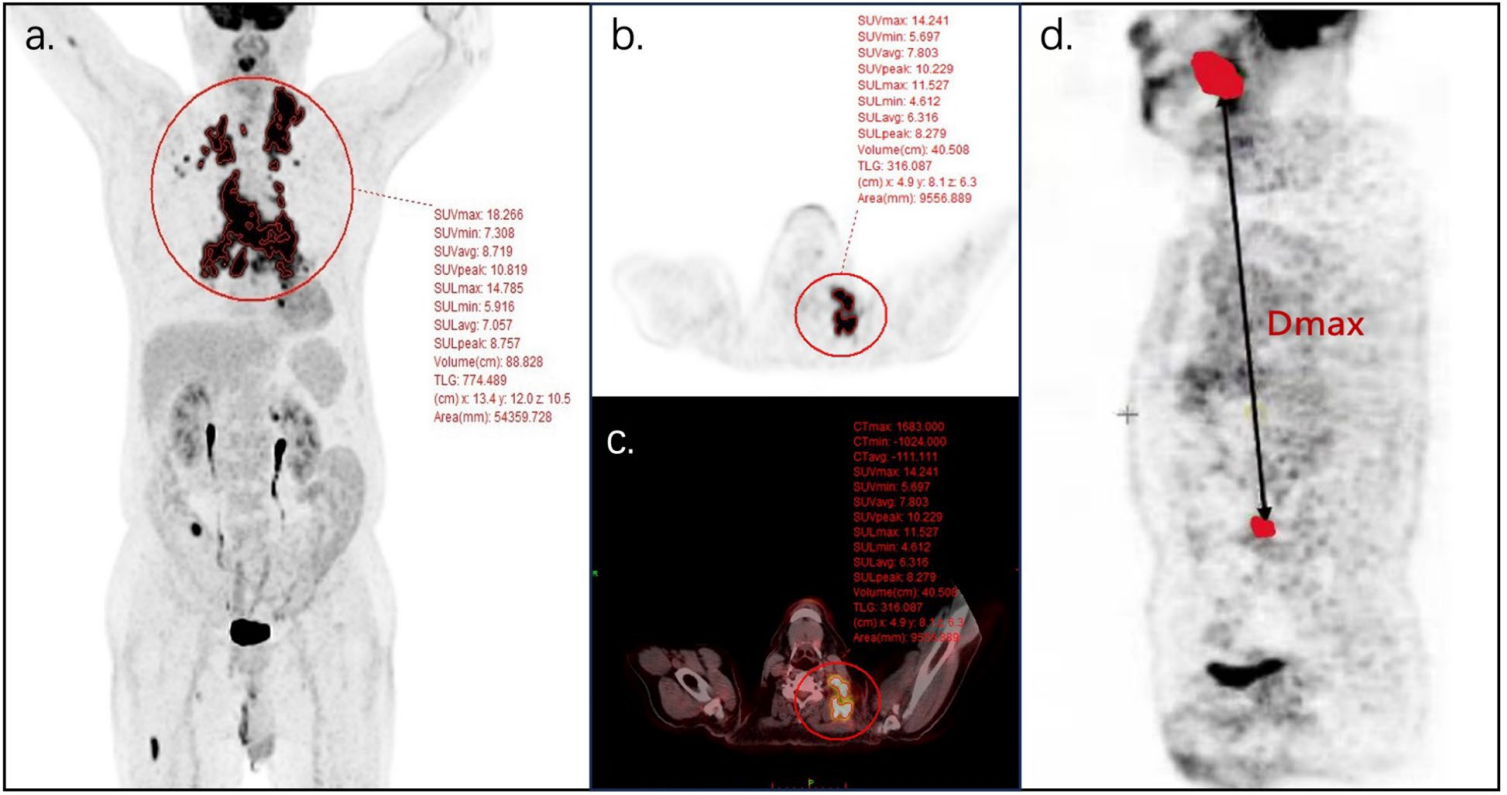
Three semiquantitative parameters in coronal (**a**) and axial coronal images(**b**, **c**) of FDG PET/CT from one patient: SUV, MTV and TLG. Dmax, the distance between two lesions that are furthest apart in sagittal images (**d**) of FDG PET/CT

The standardized uptake value (SUV), as a semiquantitative measure of FDG retention, including SUVmax, SUVmean, and SUVpeak, quantifies the ratio of radioactivity at a given image location and the whole-body injected radioactivity [13, 49]. A systematic review of 25 studies from 2011 to 2020 concluded that SUV in baseline FDG PET/CT could not predict PFS or OS in DLBCL patients [13, 48, 50, 51]. However, other FDG PET/CT indicators including MTV, TLG, and Dmax could have prognostic value (Fig. 1).

### MTV and TLG

MTV is the volume of disease contoured at a specified SUV threshold, with some semiautomated methods: a fixed SUV threshold of 2.5/4.0 g/cm^3^, 41% of SUVmax per lesion, a majority vote including voxels detected by at least 2/3 methods (MV2/3) and so on [52–54], while TLG is the sum of the products of each lesion’s MTV and SUVmean [13, 55, 56]. Some studies have compared those methods. SUV2.5 and SUV41% were recommended by El-Galaly et al., while SUV4.0 and MV2 were recommended by Barrington et al. [52, 53]. MV3 performed best in Zwezerijnen et al.’s research, with acceptable delineation in 90% of lesions and a positive agreement of 93%. It is worth noting that, in their study, delineation quality scores and agreement per method strongly depended on lesional SUV, which means that an approach that identifies the optimal delineation method per lesion as a function of tumor [^18^F]FDG uptake characteristics is required [54]. In actual situations, although MV3 performs well in some cases, the SUV2.5 and SUV41% methods are more commonly used in clinical practice due to their simplicity, ease of standardization, and extensive research support [49–51, 57].

A randomized trial demonstrated that high total metabolic tumor volume (TMTV) was significantly associated with shorter PFS and OS [58], while in the other two studies, R-CHOP resulted in significantly worse outcomes in patients with TMTV > 220 cm^3^ than in those with TMTV < 220 cm^3^ [57, 59]. With chimeric antigen receptor (CAR) T-cell therapy has emerged as an option for relapsed/ refractory (R/R) DLBCL, the high baseline TMTV has been proven as a predictor of early progression in the form of unfavorable OS [60]. Kostakoglu et al. showed that baseline TMTV and TLG were independent predictors of 4-year PFS in DLBCL patients after first-line immunochemotherapy [48]. Using a model combining baseline TLG and MTV, Ceriani et al. confirmed significantly poorer outcomes for both DLBCL and primary mediastinal B-cell lymphoma in patients at high risk of progression (*P* < 0.001), with no treatment failure in the low-risk group [56]. Most studies, however, only demonstrated that TLG was associated with survival, rather than being an independent predictor of PFS and OS, and its predictive value for DLBCL requires more in-depth research in large and multicenter studies.

MTV can serve as a single prognostic indicator and also improve the predictive reliability of prognoses based on other indicators, such as stage, IPI scores, and ECOG performance status. In 2020, Mikhaeel et al. proposed a new dynamic prognostic index for DLBCL: International Metabolic Prognostic Index (IMPI) composed of MTV, age, and stage which represents a significant advance for implementing MTV in lymphoma research [61–63]. For the patients with R/R DLBCL treated with CAR T-cell, Winkelmann et al. found that only IMPI showed a significant trend for PFS stratification (*P* = 0.030), while both IPI and IMPI didn’t show a significant association with OS after CAR T-cell [62]. Zhao et al. found that patients with low MTV had better 2-year PFS and OS than those with high MTV, especially in the low-intermediate-risk NCCN-IPI subgroup [64]. The phase III GOYA study demonstrated that patients with high TMTV and IPI had higher risks of relapse or progression than those with low TMTV and IPI (5-year PFS: 49.0% vs 74.3%) [48]. As an IPI scoring indicator, ECOG performance status has been proven to be an independent indicator of PFS and OS [57, 65]. For example, in Thieblemont et al.’s study, ECOG > 2 had a relatively high HR for PFS and OS in all three test sets [59]. Based on a positive net reclassification index for 4-year PFS and OS, Vercellino et al. concluded that a combined TMTV/ECOG variable had a higher model performance than the IPI [57], and the integrated model based on PET and tumor genotyping had a negative predictive value of 100% for disease progression or recurrence in the low-risk group.

### Dmax

Dissemination features, including the distance between two most distant lesions (Dmax_patient_) and the distance between the largest lesion and most distant lesions (Dmax_bulk_) were first proposed as DCBCL prognostic factors by Cottereau et al. in 2019 and have since been widely used [66–68]. Dmax was considered to be a better prognostic predictor for DLBCL, reflecting the extent of tumor invasion [66–69], while Dmax_patient_ and Dmax_bulk_ were negative prognostic factors for 4-year PFS (*P* < 0.001) and OS [67]. Eertink et al. also concluded that dissemination features had better predictive value than other PET parameters for 2-year progression of DLBCL [70].

Early identification of high-risk DLBCL patients who are unlikely to be cured by R-CHOP is an important step in testing alternative treatment approaches and requires a well-developed risk-scoring approach [71]. Many studies combined Dmax and MTV as complementary prognostic factors for predicting PFS and OS, reflecting tumor spread and tumor burden, respectively [66–68, 70]. In two studies involving different cohorts, a combined model based on MTV and Dmax identified significant differences in 4-year PFS and OS rates among the three groups. Specifically, this model could identify a group of patients with a poor prognosis (two risk factors) even after R-CHOP therapy, for whom clinicians might consider alternative treatment approaches [67, 71]. Standardized Dmax (SDmax) is Dmax normalized by body surface area. PFS differed significantly among three risk groups based on MTV (*P* = 0.031) and SDmax (*P* = 0.001) in high-risk (NCCN-IPI ≥ 4) and low-risk (NCCN-IPI < 4) groups [66]. Eertink et al. found that the area under the curve (AUC) for a clinical PET model based on MTV, Dmax_bulk_, SUVpeak, performance status, and age (AUC = 0.71) was significantly larger (*P* < 0.001) than that for the IPI (AUC = 0.62) [72].

### Machine learning

**Fig. 2:**
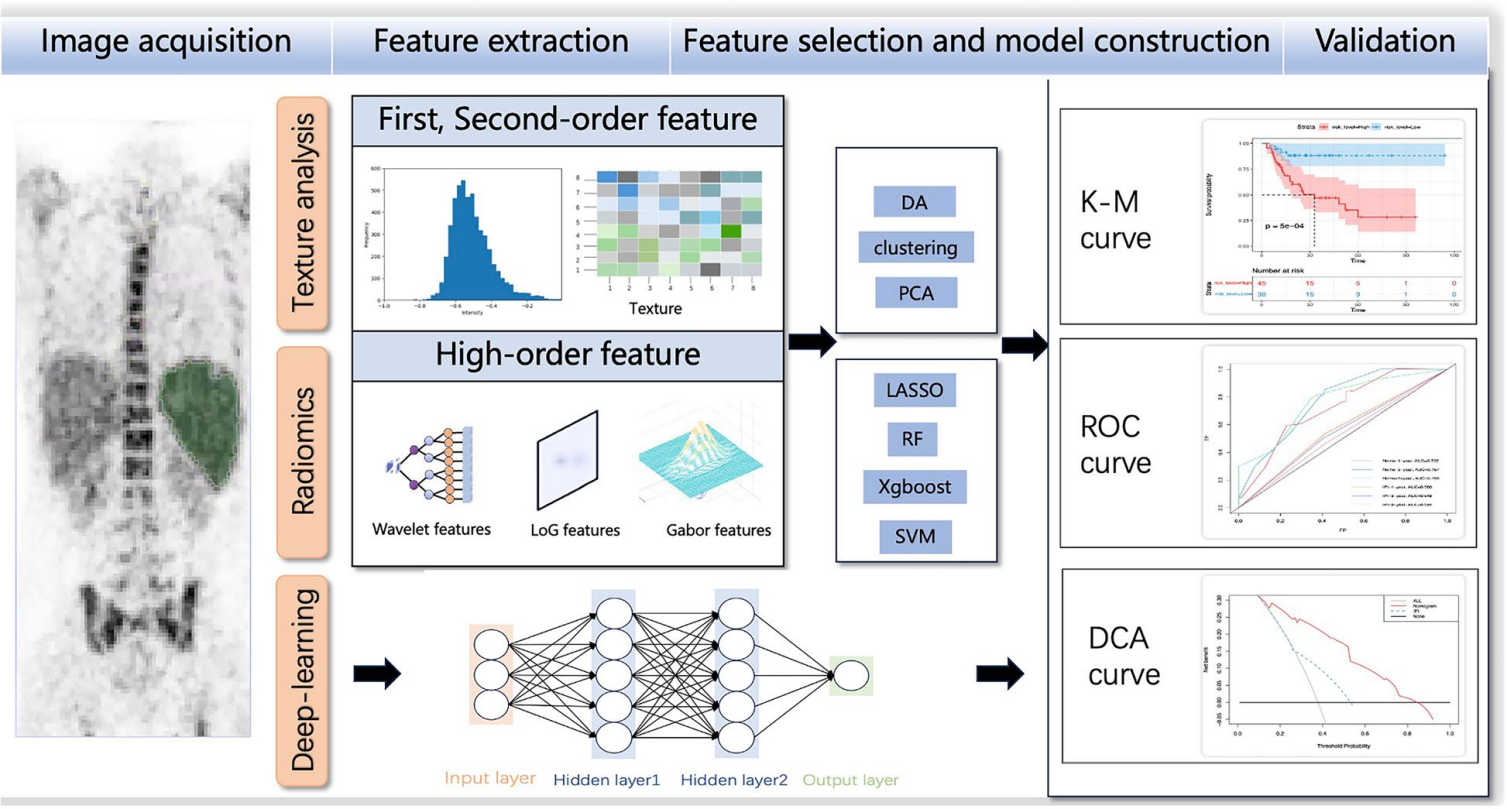
The workflow of machine learning, which includes texture analysis, radiomics, and deep learning, consists of four steps. Firstly, manually or automatically delineate the lesions to obtain the region of interest. Secondly, translate images into radiomic features. Thirdly, select features associated with prognosis for model construction. Finally, validate the predictive ability of the model internally or externally

Developments in image processing and analysis technology have led to the increasing application of computational software, fixed algorithms, and neural network models to analyze PET images to predict DLBCL progression [56, 73–76]. The main research methods currently include texture analysis, radiomics, and deep learning [73]. (Fig. 2).

### Texture analysis

Texture analysis was the first method applied in image-processing research. A combined model based on clinical and texture features had C-indices of 0.83 for PFS and 0.90 for OS, which were higher than the corresponding values of the clinical model (0.68 and 0.78) [77].

Metabolic heterogeneity (MH) is a texture features in FDG PET/CT that is calculated from the AUC of the cumulative SUV-volume histogram corresponding to the lesion with the largest MTV [56, 76]. MH can quantify the variable coefficient of glucose uptake within the tumor and reflect the inhomogeneity of the tumor microenvironment [78]. Recent studies suggested that a high MH at DLBCL diagnosis predicted a worse outcome [51, 76, 79]. Patients with large MTV and MH had a 2-year PFS rate of 42% and experienced early relapse (median PFS 11.4 months) [79]. Senjo et al. used a model integrating MH and TMTV based on two independent DLBCL cohorts to stratify patients into three groups with significantly different outcomes (5-year OS: 90.4% vs 69.5% vs 34.8%, *P* < 0.001) [76]. These studies indicated that texture analysis might help to identify high-risk patients, enabling them to be offered intensive treatment at an early stage.

### Radiomics

Radiomics conventionally constructs models by fixed algorithms using mass first-order and high-order features from images for clinical analysis. Conventional FDG PET/CT radiomics has been used in numerous lymphoma studies, and a systematic review showed that radiomics features could serve as diagnostic and prognostic indicators of lymphoma [80]. A model, which assessed by C-index and Akaike information criteria, based on WHO performance status, patient age, and radiomics provided better predictions of 2-year PFS and OS for DLBCL compared with the IPI risk score [72].

Radiomics is generally used to construct models based on combinations of features. An optimal model using clinical indicators and radiomics features predicted the 2-year time to progression of DLBCL with an AUC of 0.79 [68]. A wavelet transform model incorporating clinical indicators and FDG PET/CT radiomics yielded a higher AUC (0.75) than a model based solely on MTV (0.67) to predict 2-year event-free survival in patients with DLBCL [75]. A combined model of DLBCL progression based on metabolic metrics, clinical risk factors, and FDG PET/CT radiomics was superior to the single model and provided high C-indices for both the training set (PFS 0.825, OS 0.834) and validation set (PFS 0.831, OS 0.877) [74, 81]. A model combining BCL-6 and radiomics features dimensionally reduced using linear discriminant analysis had high predictive efficiency for DLBCL (AUC = 0.904, accuracy 90%, sensitivity 100%, specificity 80%) [82]. By comparing the time-dependent ROC curves, a nomogram including blood platelet count, sex, and radiomics score (Rad-scores) had been proven to provide a better recurrence risk assessment [83], and Zhao et al. also proved that a combination of different classifiers yielded a higher AUC for DLBCL prognosis than a single classifier [84].

Despite the promise of PET radiomics, some challenges still need to be addressed to improve model reliability and interpretability [80, 85]. However, radiomics models could be successfully used in DLBCL clinical settings if more robust prognostic models can be established using big data from multicenter studies.

### Deep learning

Deep learning neural networks, especially convolutional neural networks (CNN), have been widely used to identify, segment and tumors, extract features and predict outcomes. [73, 86–89].

It’s crucial to identify early patients with bone marrow (BM) involvement since BM lymphoma invasion is a sign of advanced disease [90]. The BM lesions obtained from FDG PET/CT through the method of manual detection, radiomics or deep-learning combined with the results of bone marrow biopsy, could all improve the detection of BM involvement in patients with DLBCL and provide more accurate prognoses [26, 91–94]. For example, Jemaa et al. proved that patients with both positive biopsy and PET results, analyzed using a deep learning algorithm, have the worst prognosis compared to those with both negative results (2-year PFS: 62% vs. 72%) [26].

With the DLBCL patients as the training set, on the follicular lymphoma test set, a novel cascaded 2D to 3D CNN architecture produced a Dice Similarity Coefficient (DSC) of 0.886 and a voxel level sensitivity of 92.6% in identifying and segmenting tumors [87]. Weisman et al. implemented a 3D, multiresolution pathway CNN, DeepMedic to automatically detect lymph nodes involved in lymphoma and achieved a true-positive rate (TPR) of 85% [89]. Based on these predicted tumor mask, the automatic calculated TMTV could yield very precise estimates in Jemaa et al.’s study with Spearman’s correlations respectively of 0.97 compared with ground truth and predict the outcomes of DLBCL in Capobianco et al.’s study with 4-y OS rates were 90% and 74% for the low- and high-TMTV groups (optimal TMTV cutoffs: 148 cm^3^) [86, 87].

Haggstrom et al.’s ResNet34-based deep learning model could distinguish lymphoma patients, including DLBCL with and without hypermetabolic tumour sites, for binary classification (Deauville 1-3 vs 4-5), with AUC, accuracy, and sensitivity all exceeding 0.9 [95]. Jemaa et al.’s deep learning-based algorithm for automated metabolic response assessment in Lugano had strong prognostic value for outcomes. In three trials, there was a trend toward greater accuracy for risk of death than adjudicated radiologic responses (hazard ratio for end of treatment CMR of 0.123, 0.054, and 0.205 vs 0.226, 0.292, and 0.272, respectively) [96]. Deep learning for automated treatment response assessments in DLBCL would eventually change workflows and labor and resource allocation in clinical research and practice [97], as end-of-treatment PET response had been shown to be prognostic for OS [98–100].

Deep learning models have been applied in numerous diseases, including lymphoma, breast cancer, rectal cancer, and nasopharyngeal carcinoma [101–103]. Multiparametric models based on patient age, Ann Arbor stage, SUVmax, TMTV, and deep learning scores obtained from VGG19 and DenseNet121 networks were built to predict DLBCL prognosis, with C-indices 0.866 for PFS and 0.835 for OS, and were verified by C-index in external validation cohorts [104]. A deep learning model based on interim FDG PET/CT images showed good performance in a test cohort (AUC = 0.926) and external datasets (AUC = 0.925) for directing individualized clinical treatment of DLBCL patients [105].

## Discussion

Comparing studies can summarize ways to boost research reliability. For multicenter radiomics studies, which are reliable than single-center studies, it is necessary to use the Combat method to assess the differences between various scanners. Most literature used ROC to rate models. Some of them implemented DeLong for AUC, assessing differences like sample size more rigorously. In addition to comparing the prognostic capabilities of models containing different indicators, there could be more articles comparing the models built using different machine learning methods, such as logistic regression with Least Absolute Shrinkage and Selection Operator (LASSO), ridge and elasticnet penalties, support vector machine and random forest.

However, the clinical adoption of artificial intelligence (AI) methods has been hindered by the lack of interpretability and generalizability, so increasing the interpretability of AI algorithms and creating a supervised deep-learning system for medical imaging based on a large, labeled dataset could gain the confidence of doctors and patients [106]. As the first step of the research, most studies used a single method to segment the lymphoma lesions. The heterogeneity in DLBCL lesion tracer uptake means a one-size-fits-all segmentation approach might not be ideal for all patients [68, 89]. Another key limitation is studies comparing human and AI diagnostics often lack real-world clinical context, relying solely on images without considering patient histories or additional data. This often increases the difficulty of the diagnostic task for the human reader [107].

## Conclusion

DLBCL is a heterogeneous disease with series of baseline IPI-based scoring standards, which have been iteratively developed to address the heterogeneity of clinical outcomes. Molecular characteristics have been included to improve predictions of DLBCL progression and identify novel biological targets. Various baseline FDG PET/CT parameters, including MTV, TLG, and Dmax, have been used to construct machine learning models, but baseline radiomics and deep learning models that can predict the outcome of DLBCL remain in their infancy. Further development of AI technology will provide better predictive models based on big data from multicenter studies.

## Data Availability

No datasets were generated or analysed during the current study.

## Abbreviations

aa-IPI: Age-adjusted IPI
ABC: Activated B-cell-like
AI: Artificial intelligence
AUC: Area under the curve
BM: Bone marrow
CAR: Chimeric antigen receptor
CCND2: Cyclin D2
CMR: Complete metabolic response
CNN: Convolutional neural networks
CNS-IPI: Central nervous system IPI
DA: Domain adaptation
DCA: Decision curve analysis
DLBCL: Diffuse large B-cell lymphoma
ECOG: Eastern Cooperative Oncology Group
FDG: Fluorodeoxyglucose
GCB: Germinal-center B-cell-like
IBPS: Inflammation-Based Prognosis Score
IMPI: International Metabolic Prognostic Index
IPI: International Prognostic Index
IRF4: Interferonregulatoryfactor4
K-M: Kaplan-meier
MH: Metabolic heterogeneity
MTV: Metabolic tumor volume
MUM1: Multiplemyelomaoncogene1
MV2: Voxels detected by at least 2 methods
MV3: Voxels detected by at least 3 methods
NCCN-IPI: National Comprehensive Cancer Network IPI
OS: Overall survival
PCA: Principal component analysis
PET/CT: Positron emission tomography computed tomography
PFS: Progression-free survival
RF: Random forest
ROC: Receiver operating characteristic
R/R DLBCL: Relapsed/ refractory DLBCL
R-IPI: Revised IPI
SUV: Standardized uptake value
SVM: Support vector machine
TLG: Total lesion glycolysis
WHO: World Health Organization
